# Global evaluation of key factors influencing nitrogen fertilization efficiency in wheat: a recent meta-analysis (2000-2022)

**DOI:** 10.3389/fpls.2023.1272098

**Published:** 2023-10-30

**Authors:** Solomon Yokamo, Muhammad Irfan, Weiwei Huan, Bin Wang, Yiliu Wang, Muhammad Ishfaq, Diajun Lu, Xiaoqin Chen, Qiuliang Cai, Huoyan Wang

**Affiliations:** ^1^ State Key Laboratory of Soil and Sustainable Agriculture, Institute of Soil Science, Chinese Academy of Sciences, Nanjing, China; ^2^ University of Chinese Academy of Sciences, Beijing, China; ^3^ Department of Plant Nutrition, College of Resources and Environmental Sciences; The State Key Laboratory of Nutrient Use and Management (SKL-NUM), Ministry of Education, China Agricultural University, Beijing, China; ^4^ Guangxi Key Laboratory of Biology for Mango, College of Agriculture and Food Engineering, Baise University, Baise, China

**Keywords:** fertilizer management, meta-analysis, nitrogen rate, recovery efficiency, split application, wheat yield

## Abstract

Improving nitrogen use efficiency (NUE) without compromising yield remains a crucial agroecological challenge in theory and practice. Some meta-analyses conducted in recent years investigated the impact of nitrogen (N) fertilizer on crop yield and gaseous emissions, but most are region-specific and focused on N sources and application methods. However, various factors affecting yield and N fertilizer efficiency in wheat crops on a global scale are not extensively studied, thus highlighting the need for a comprehensive meta-analysis. Using 109 peer-reviewed research studies (published between 2000 and 2022) from 156 experimental sites (covering 36.8, 38.6 and 24.6% of coarse, medium, and fine texture soils, respectively), we conducted a global meta-analysis to elucidate suitable N management practices and the key factors influencing N fertilization efficiency in wheat as a function of yield and recovery efficiency and also explained future perspectives for efficient N management in wheat crop. Overall, N fertilization had a significant impact on wheat yield. A curvilinear relationship was found between N rates and grain yield, whereas maximum yield improvement was illustrated at 150-300 kg N ha^-1^. In addition, N increased yield by 92.18% under direct soil incorporation, 87.55% under combined chemical and organic fertilizers application, and 72.86% under split application. Site-specific covariates (climatic conditions and soil properties) had a pronounced impact on N fertilization efficiency. A significantly higher yield response was observed in regions with MAP > 800 mm, and where MAT remained < 15 °C. Additionally, the highest yield response was observed with initial AN, AP and AK concentrations at < 20, < 10 and 100-150 mg kg^-1^, respectively, and yield response considerably declined with increasing these threshold values. Nevertheless, regression analysis revealed a declining trend in N recovery efficiency (REN) and the addition of N in already fertile soils may affect plant uptake and RE. Global REN in wheat remained at 49.78% and followed a negative trend with the further increase of N supply and improvement in soil properties. Finally, an advanced N management approach such as “*root zone targeted fertilization*” is suggested to reduce fertilizer application rate and save time and labor costs while achieving high yield and NUE.

## 1 Introduction

The ever-mounting world food demand drives agriculture toward the intensification of crop and animal production (Tilman et al., 2011; Zhang et al., 2020). This is directly related to the enormous use of agricultural resources, particularly N fertilizer. Nitrogen, being an essential macronutrient, is the most crucial nutrient for plant growth and development (Irfan et al., 2021; Kubar et al., 2021). It plays a significant role in feeding nearly half of the world’s population (Zhang et al., 2015; Liu et al., 2016). During the past few decades, an enormous increase in fertilizer N use in arable soils has been reported to enhance global food production (Sun et al., 2020). For example, world cereal production has increased by 3.4-fold (340%) during the past 60 years (1961-2020) ascribing to a 9.45-fold increase in N consumption in cereal crops (FAOSTAT, 2021; Yokamo et al., 2022a). As a result, over-application is becoming a common practice, particularly in countries like China that have a high level of intensive production systems (Zhang et al., 2015; Jiang et al., 2019). However, excessive N application does not always translate into a continuous improvement in crop productivity, rather it leads to several environmental and ecological problems (Zhang et al., 2012; Zhang et al., 2019; Song et al., 2023) such as air pollution, soil acidification, and eutrophication of water bodies (Chen et al., 2014; Ren et al., 2022) and undermine the sustainability of food and energy production. Nevertheless, the optimum use of N fertilizer has been recognized as an important factor to maintain crop yield, minimizing ammonia (NH_3_) losses and greenhouse gas (GHG) emissions (Dungan et al., 2021).

Improving NUE in agroecosystems has significant agronomic, economic, environmental, and health implications (Fageria et al., 2015; Zhang et al., 2015; Dimkpa et al., 2020). The NUE (i.e., the fraction of N input harvested as product), is an established metric used as an indicator for evaluating a crop’s ability to convert available soil N into economic yield (Fageria et al., 2015; Congreves et al., 2021). Although N is the key element in boosting world food production, its excessive application beyond the crop’s requirement lower NUE and results in adverse environmental consequences (Fageria et al., 2015; Khatun et al., 2015). An earlier study reported that the global N-recovery efficiency is below 50% (Ladha et al., 2005). Other studies also reported that a global NUE in cereal crops was 33% as reported in 1999 (Raun and Johnson, 1999) which increased up to 35% in 2015 (Omara et al., 2019) indicating that more than half of the applied N is lost to the environment through different N-loss mechanisms. This not only pollutes surface-ground water and the atmosphere but also reduces economic benefits (Li et al., 2021). However, the NUE of a cropping system can therefore be increased by achieving greater uptake efficiency from applied N inputs, and hence reducing the N loss from soil organic and inorganic N pools, or both (Cassman et al., 2002; Belete et al., 2018).

A comprehensive understanding and knowledge-based N management practices are imperative to design and develop new paradigms to explore optimum N supply rate that will improve crop yield, NUE, and ecosystem services while reducing potential N losses to the environment (Chen et al., 2017; Song et al., 2022a). To solve the aforementioned conundrum, several fertilizer management strategies have been suggested including enhanced efficient fertilizers (EEFs) (Xia et al., 2017; Zhu et al., 2020), integrated nutrient management (INM) (Zhang et al., 2012; Selim, 2020), sole or integrated application of organic inputs with synthetic fertilizer (Wang et al., 2019; Cen et al., 2020; Yang et al., 2020), and split N application (Belete et al., 2018). The EEFs are used to delay N transformation processes and/or slow the N release pattern through coated materials or inhibitors to better synchronize N release and plant uptake, hence enhancing NUE and reducing losses (Tao et al., 2021). Split-surface broadcasting (SSB) of N is a widely adopted fertilizer management approach all over the world. It is one of the methods to enhance NUE by minimizing the N losses via NH_3_ volatilization, leaching, and runoff. In addition, determining the right N application timing is a decisive approach in gaining high yield and high efficiency. Several studies reported a positive effect of SSB on improving grain yield. However, some studies criticized that split application is not effective in increasing yield but rather exacerbates N losses. This is mainly because urea applied by this method may result in quick hydrolysis and then be prone to loss from the soil-plant system due to its distance from the root zone (Irfan et al., 2021; Song et al., 2023).

Meta-analysis is a quantitative statistical analysis technique that syndicates the result from different individual studies into one report to obtain a precise estimate of the effect (Adams et al., 1997; Bohoussou et al., 2022). Recent years have seen a rise in the use of meta-analysis to investigate the impact of fertilizer on crop yield and gaseous emissions. For instance, an earlier study assessed management-induced changes in N partial factor productivity and identified relevant strategies for winter wheat from data published between 1979-2016 (Liu et al., 2020). However, this study is region-specific (i.e., it focused only on North China Plain). Although another meta-analysis study reported factors affecting the nitrogen recovery efficiency of the three crops, i.e., rice, wheat, and maize by using data published until 2020 (Yu et al., 2022a), however, factors affecting the productivity of these crops were not detailed. Moreover, other studies were focused on specific N sources and application methods, thus highlighting the need for a comprehensive meta-analysis. Also, factors affecting wheat yield response and N recovery efficiency on a global scale were not extensively studied. Therefore, to highlight suitable N management practices and factors affecting their efficacy, we conducted the first comprehensive global meta-analysis to assess the key factors influencing N fertilization efficiency in wheat and accounting for the differences in REN and yield. By compiling 109 original research papers that were published over 22 years (2000 to 2022), the main objectives of the current study were (i) to evaluate the wheat yield response and REN to N fertilization and (ii) to assess factors affecting the magnitude of response such as N management practices, climate, and initial soil properties. Moreover, we also highlighted some future perspectives as well as further research directions to enhance NUE in wheat crops.

## 2 Materials and methods

### 2.1 Literature search and data collection

An extensive literature survey was conducted from the Web of Science (https://www.webofscience.com//), Google Scholar (https://scholar.google.com), and ResearchGate (https://www.researchgate.net). Different combinations of search strings were used to access the published papers as: (N fertilizer* OR urea* OR organic N* OR slow-release urea* OR N split application* OR N sources* OR N rate*) AND (wheat* OR N recovery efficiency* OR N use efficiency). Studies were scrutinized to include in the meta-analysis if they met the following quality criteria: i) Only field experiments (pot, greenhouse, or laboratory incubation experiments were excluded), ii) Experiments that were replicated three times or more, iii) Experiments that compared experimental unit (N) and control (without N) but all management practices were the same, iv) Experiments that reported at least wheat grain yield, N recovery efficiency, or aboveground N uptake, and v) Studies that reported other relevant information precisely such as N management practice, climate, and initial soil properties.

**Figure 1:**
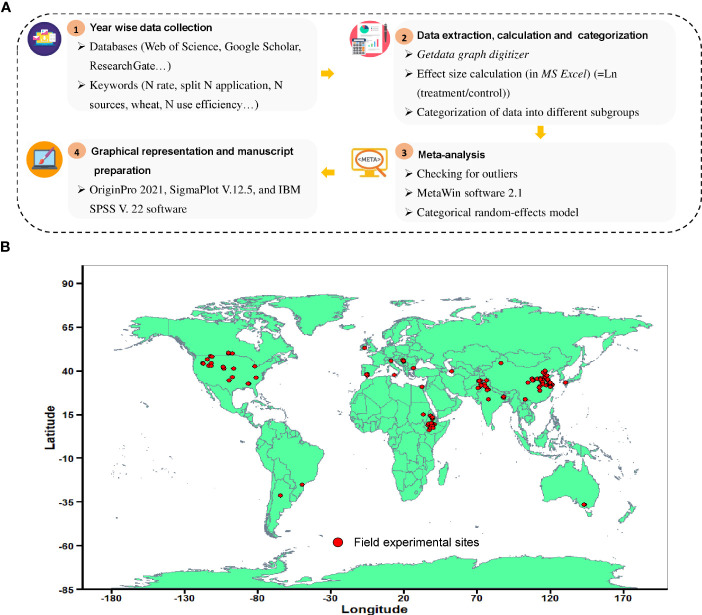
**(A)** Stepwise schematic description highlighting the summary of this study and **(B)** the geographical distributions of the field experimental sites.

The data presented in the figures were digitized to obtain a numerical value by using the software “Get-Data Graph Digitizer” (http://getdatagraphdigitizer.com/). Soil organic carbon (SOC) was converted into soil organic matter (SOM) by multiplying the former by the conversion factor of 1.724. The soil parameters (topmost) reported in percent were changed to their respective units of g kg^-1^ and mg kg^-1^. Overall, 1,995 paired observations from 109 studies for grain yield and 833 observations from 52 studies for REN have fulfilled our specific criteria for this study (see **Figure S1**; **Table S1**). Information on N management (i.e., application rate, source, timing, and application method), climate (i.e., season, MAT, and MAP), and initial soil properties (i.e. texture, pH, SOM, total nitrogen (TN), available nitrogen (AN), available phosphorous (AP)and available potassium (AK)), and crop growing seasons were included and used to further specify statistical relationships between N fertilization and crop yield and REN. The stepwise schematic description of the summary of this study and the geographic distribution of the field experiments has been illustrated in **Figures 1A, B**, respectively.

Grain yield was directly collected from the studies. Among the two approaches to quantify N use efficiency (the difference approach and N balance approach), we have considered a REN value calculated through the ‘‘N-difference’’ method, since this is most pertinent to agricultural practices (You et al., 2023). The N-difference method is based on the difference in N-uptake between a crop that receives a specific amount of N and a reference plot without applied N (Cassman et al., 2002). It is a suitable index for research contexts because: i) it considers background soil N contents by accounting for N uptake or production in unfertilized plots, and ii) it is a simple and cost-effective method (Elrys et al., 2022). The majority of studies reported N use efficiency based on the total aboveground N uptake in fertilized and unfertilized plots; however, there is a lack of information on N deposition and fixation needed to determine total N inputs. Thus, in the occasion that the studies did not report REN, we computed it from total aboveground N uptake using equation (1) below.


(1) $$\text{REN}=\left(\frac{{\text{NU}}_{\text{trt}}-{\text{NU}}_{\text{ck }}}{{\text{F}}_{\text{N}}}\right)*100$$

Agronomic N use efficiency (AE_N_) and partial factor productivity (PFP) were calculated as:


(2) $${\text{AE}}_{\text{N}}=\left(\frac{{\text{GY}}_{\text{trt}}-{\text{GY}}_{\text{ck }}}{{\text{F}}_{\text{N}}}\right)$$

(3) $$\text{PFP}=\frac{{\text{GY}}_{\text{trt}}}{{\text{F}}_{\text{N}}}$$

Where NU_trt_ and NU_ck_ represent total aboveground N uptake in N applied and control plots, respectively; GY_trt_ and GY_ck_ denote grain yield in N applied and control plots, respectively, and F_N_ denotes the amount of N fertilizer applied.

### 2.2 Data categorization

The categories were chosen to involve sufficient measurements and publications for meta-analysis. Chemical N sources include urea, ammonium nitrate, ammonium sulfate, and diammonium phosphate. All controlled or slow-release fertilizers (CRF/SRF) were categorized under release fertilizer. All manure sources and crop residues were categorized under organic fertilizer. The N application method was categorized as incorporation within top layer soils (incorporation), broadcasting, and placement (i.e., application within seed rows, side dressing and banding below the soil surface at different depths, banding below the seed, injection, and drilling by the planter directly into the seed row). Soil textural classes were grouped into three categories based on the USDA textural classes: coarse (sand, loam, silt, sandy loam, loamy sand, and silt loam), medium (clay loam, silty clay loam, and sandy clay loam) and fine (clay, silty clay, and sandy clay) as reported in Li et al. (2018), which covers 36.8, 38.6 and 24.6%, respectively. Moreover, if the sub-category had <10 observations, they were not considered in our analysis. Detailed information on the categorization of explanatory variables with their sample size and bootstrap confidence intervals (CIs) is presented in **Table 1**.

**Table 1:** A list of explanatory variables, sample size (*n*), and bootstrap confidence intervals (CIs).

Items	Variables	Groups	*n*	Bootstrap CI
Climate conditions	Mean annual temperature (MAT, °C)	< 15	369	1.7269 to 1.8698
		> 15	343	1.5777 to 1.7861
	Mean annual precipitation (MAP, mm)	< 800	476	1.6038 to 1.7435
		> 800	258	1.7549 to 2.0416
	Season (wheat type)	Winter wheat	846	1.7460 to 1.8592
		Spring wheat	514	1.2237 to 1.3081
N management	N rate (kg ha^-1^)	<150	1145	1.4798 to 1.5414
		150-300	770	1.7498 to 1.8890
		>300	89	1.5503 to 1.8868
	N timing (frequency)	One time	495	1.4294 to 1.5612
		Split (≥2)	1231	1.6869 to 1.7764
	N source	Chemical	1837	1.5692 to 1.6434
		Organic	26	1.3272 to 1.5888
		Mixture	108	1.7653 to 2.0024
	Method of first N application	Placement	296	1.5973 to 1.5120
		Broadcasting	356	1.5357 to 1.4186
		Incorporation	157	2.1475 to 1.7283
Initial soil properties	Soil texture	Coarse	295	1.6967 to 1.8367
		Medium	310	1.7187 to 1.9839
		Fine	197	1.6232 to 1.8038
	Soil pH	Acidic (<6.5)	323	1.5299 to 1.6709
		Neutral (6.5-7.5)	317	1.687 to 1.9059
		Alkaline (>7.5)	731	1.4706 to 1.6896
	SOM (g kg^-1^)	<10	282	1.5978 to 1.9478
		10-20	568	1.6067 to 1.7239
		20-35	275	67.51 to 101.170
		>35	65	49.701 to 77.470
	TN (g kg^-1^)	<1	504	1.8767 to 2.0531
		≥1	332	1.6546 to 1.8620
	AN (mg kg^-1^)	<20	137	1.7925 to 1.9708
		20-40	176	1.6930 to 1.9601
		>40	173	1.6327 to 1.8709
	AP (mg kg^-1^)	<10	419	1.6306 to 1.8185
		10-20	432	1.5184 to 1.6167
		>20	326	1.5016 to 1.6379
	AK (mg kg^-1^)	<100	201	1.560 to 1.7049
		100-150	201	1.8376 to 2.0449
		>150	330	1.3962 to 1.5062

### 2.3 Meta-analysis

This meta-analysis was performed to evaluate the effects of N management on wheat yield and REN and the subsequent explanatory variables affecting the magnitude of yield response and REN across the study. Mean annual temperature (MAT), mean annual precipitation (MAP), season, N application rate, source, timing and first N application methods, and initial soil properties within a single publication were considered as explanatory variables, whereas grain yield and REN were considered as dependent variables. The NUE was directly used in the analysis after removing the outliers which are identified by the boxplot with SPSS V.22. In this study, a nonparametric weighting function was used to process the data due to only a few studies (<30%) were reported a standard error (Adams et al., 1997). To avoid bias due to site factors, a particular study that included different experiments were treated independently and described in the study as separate data units. As a result, the weight of the effect size was calculated as follows:


(4) $$\text{Weight}=\frac{\text{Nt x Nc}}{\text{Nt}+\text{Nc}}$$

Where, N_t_ and N_c_ indicate the number of repetitions of the treatment and control, respectively.

A random-effects model was used to determine the effects of N-induced effects on grain yield. Therefore, the natural logarithm of the response ratio (ln*RR*) was calculated as the effect size (Hedges et al., 1999), and the *RR* distribution of wheat yield response to N fertilization was illustrated in the **Supplementary Materials** (**Figure S2**).


(5) $$\text{ln}RR=ln\left(\frac{\text{Xt}}{\text{Xc}}\right)=ln\left(\text{Xt}\right)-ln\left(\text{Xc}\right)$$

For a straightforward visualization and ease of interpretation, all results were back-transformed to a percentage change using the following formula as indicated in (Pittelkow et al., 2015):


(6) $$\text{Percentage change }\left(\%\right)=\left({\text{exp}}^{\text{lnE}}-1\right)*100$$

### 2.4 Statistical analysis

Statistical processing of the data was performed by adopting a categorical random effect model and a bootstrapping procedure (4999 iterations) through MetaWin software 2.1. All the illustrations were drawn using Sigma Plot V.12.5, Origin Pro 2021, and IBM SPSS Statistics V.22 software. Differences between treatments and controls were considered significant when its back-transformed mean effect size and 95% CIs did not overlap with zero and non-significant when it crosses the line of zero effect.

## 3 Results

### 3.1 Wheat yield response to N fertilization: effect of N management practices

**Figure 2:**
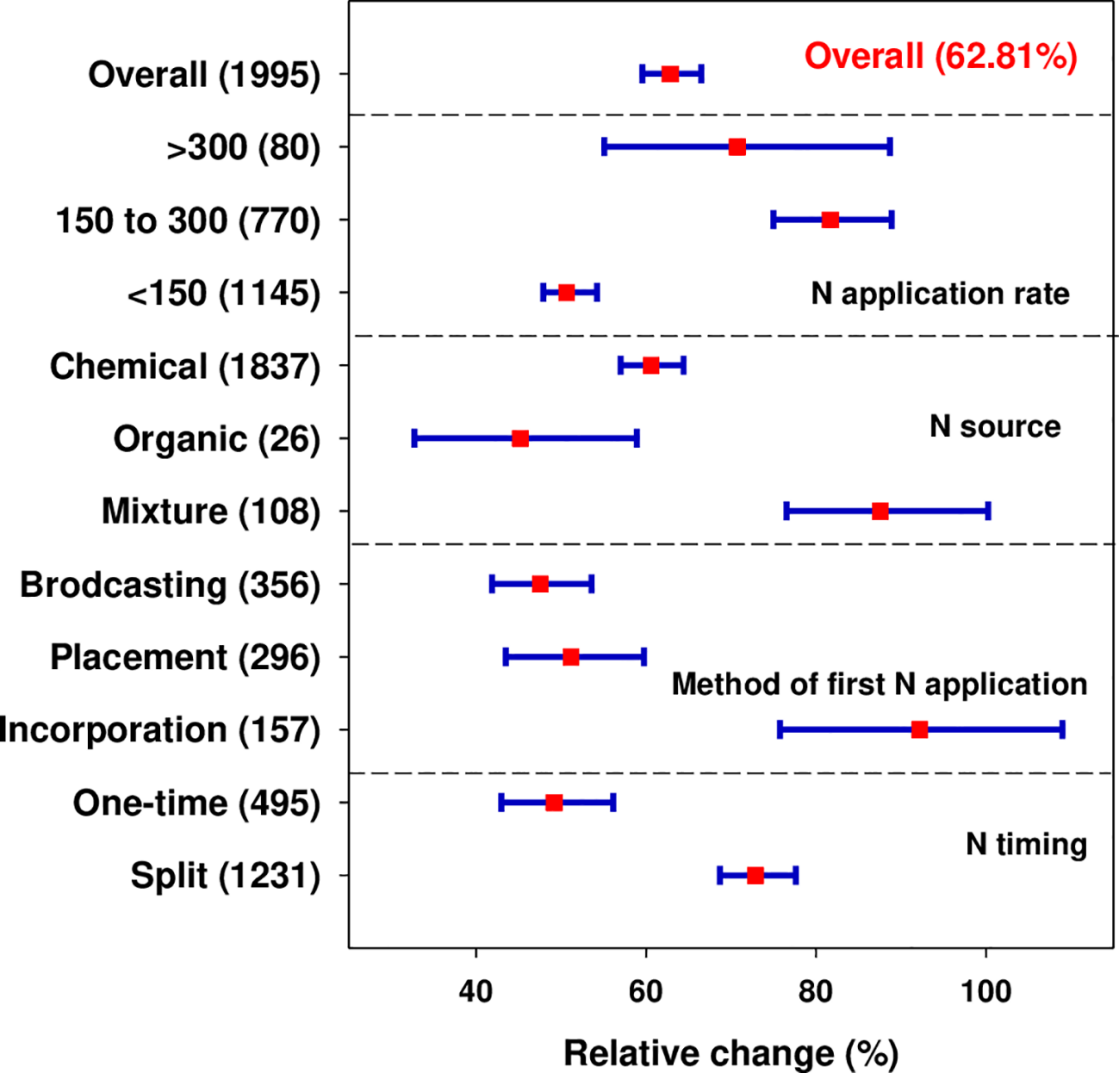
Effect of N fertilization on wheat yield as affected by N application rates, sources, application methods, and timing/frequency. ‘One-time’ is one-time fertilizer N application during the whole wheat plant growing season regardless of application methods while split represents N application of ≥2. The N application rate: low (<150 kg ha^-1^), medium (150-300 kg ha^-1^) and high (>300 kg ha^-1^). Error bars represent a mean value at 95% confidence intervals (CIs). The number of observations is indicated in parentheses.

**Figure 3:**
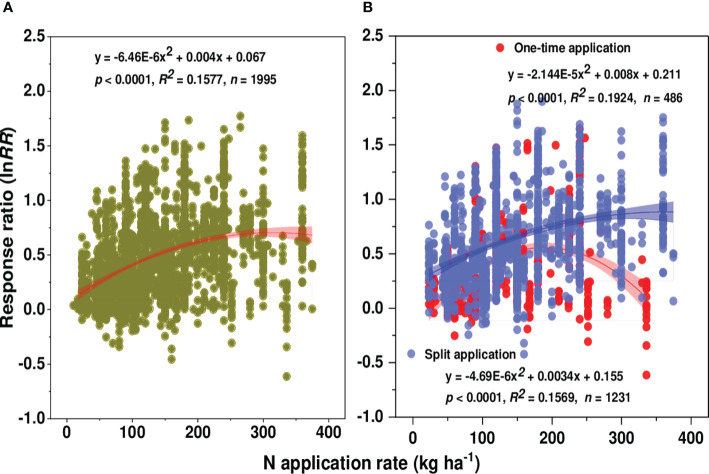
Relationship between N rate and the natural log of response ratio (ln*RR*), **(A)** overall response and **(B)** at one-time vs split N application method.

The results of our meta-analysis showed that across all studies and relative to control, N fertilization significantly increased wheat grain yield by 62.81% (**Figure 2**). A strong regression relationship was observed between yield and N application rates. Wheat yield increased curvilinearly with increasing N supply rate as illustrated in **Figure 3A**. Four N management factors (i.e., N application rate, source, method of first N application, and timing/frequency) were illustrated in **Figure 2**. Under different N rates, the highest yield increase over control (81.68%) was observed at the N rate of 150-300 kg ha^-1^, nevertheless further increase or decrease in N above or below this level considerably reduced wheat yield. Generally, wheat yield increased initially and after reaching the threshold at a certain N supply rate, yield started to decline despite increasing N application rates. The highest yield response was observed when N was incorporated within the soil, which lifted the wheat yield by 92.18% over broadcasting (47.49%) and placement (51.12%). However, the effect of N fertilization on grain yield did not differ significantly between broadcasting and placement.

The effect of N timing/frequency on wheat yield remained significant. The yield response was higher (72.86%) with the split application than with the one-time application (49.15%) regardless of application methods. Moreover, the positive response of N fertilization varied with the number of splits; increased with 3-split (84.66%) followed by ≥4-split (80.85%) and decreased with 2-split (69.28%) (**Figure S3**). We conducted a regression analysis of the natural log of yield response and N rate based on one-time and split N application and found a quadratic relationship (*R* ^2^ for split = 0.1569 and one-time = 0.1924) as shown in **Figure 3B**. The effect of N fertilization on wheat yield is significantly affected by N sources as shown in **Figure 2**. A positive and significantly higher yield response was observed with the combined application of organic and chemical N application regardless of substitution ratio (87.55%) than chemical (60.56%) and organic N alone (45.16%). Detailed categorization of different chemical N sources has been illustrated in **Figure S4**. The yield response was greater (131.44%) when urea was applied with release fertilizer (urea + release fertilizer) followed by the sole application of release fertilizer (72.95%). Contrarily, the response was lowest when N was applied in ammonium nitrate forms (35.56%) followed by ammonium sulphate (27.75%).

### 3.2 Wheat yield response to N fertilization: effect of climatic conditions

**Figure 4:**
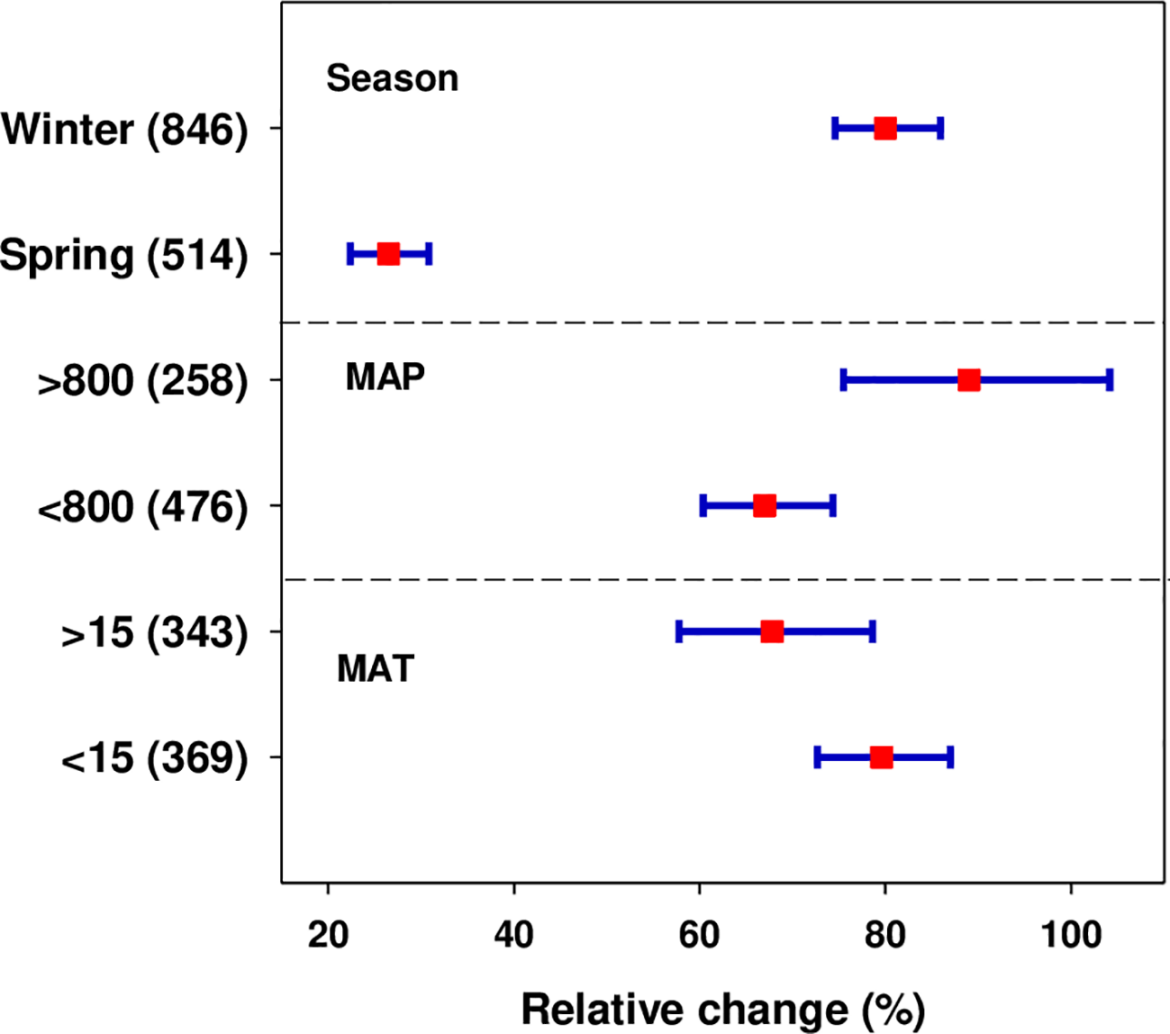
Effect of N fertilization on wheat yield as affected by climatic conditions (season (spring or winter), MAT: mean annual temperature (°C) and MAP: mean annual precipitation (mm)). Error bars represent a mean value at 95% CIs. Numbers in parentheses indicate the number of observations.

The effect of N fertilization on wheat yield was significantly affected by seasonal variation (wheat type), MAT, and MAP as presented in **Figure 4**. Winter wheat had a profoundly higher yield response (80.03%) than spring wheat (26.49%). We found that the wheat yield response to N application was strongly dependent on climate conditions. Temperature is an important yield-determining factor so its deviation from optimum range because of climate change or other factors may negatively impact crop productivity. The result revealed that a significant yield response of 79.60% and 67.81% was observed when MAT remained < 15°C and > 15°C, respectively. The amount, intensity, and pattern of rainfall distribution across the seasons considerably influence the grain yield response to N fertilization. Plant growth and productivity are closely linked to the amount of moisture available during the growing season (intra-season). Our analysis revealed that wheat yield to N application increased in regions receiving MAP > 800 mm (88.99%) than in regions with MAP < 800 mm (67.02%) (**Figure 4**).

### 3.3 Wheat yield response to N fertilization: effect of initial soil properties

**Figure 5:**
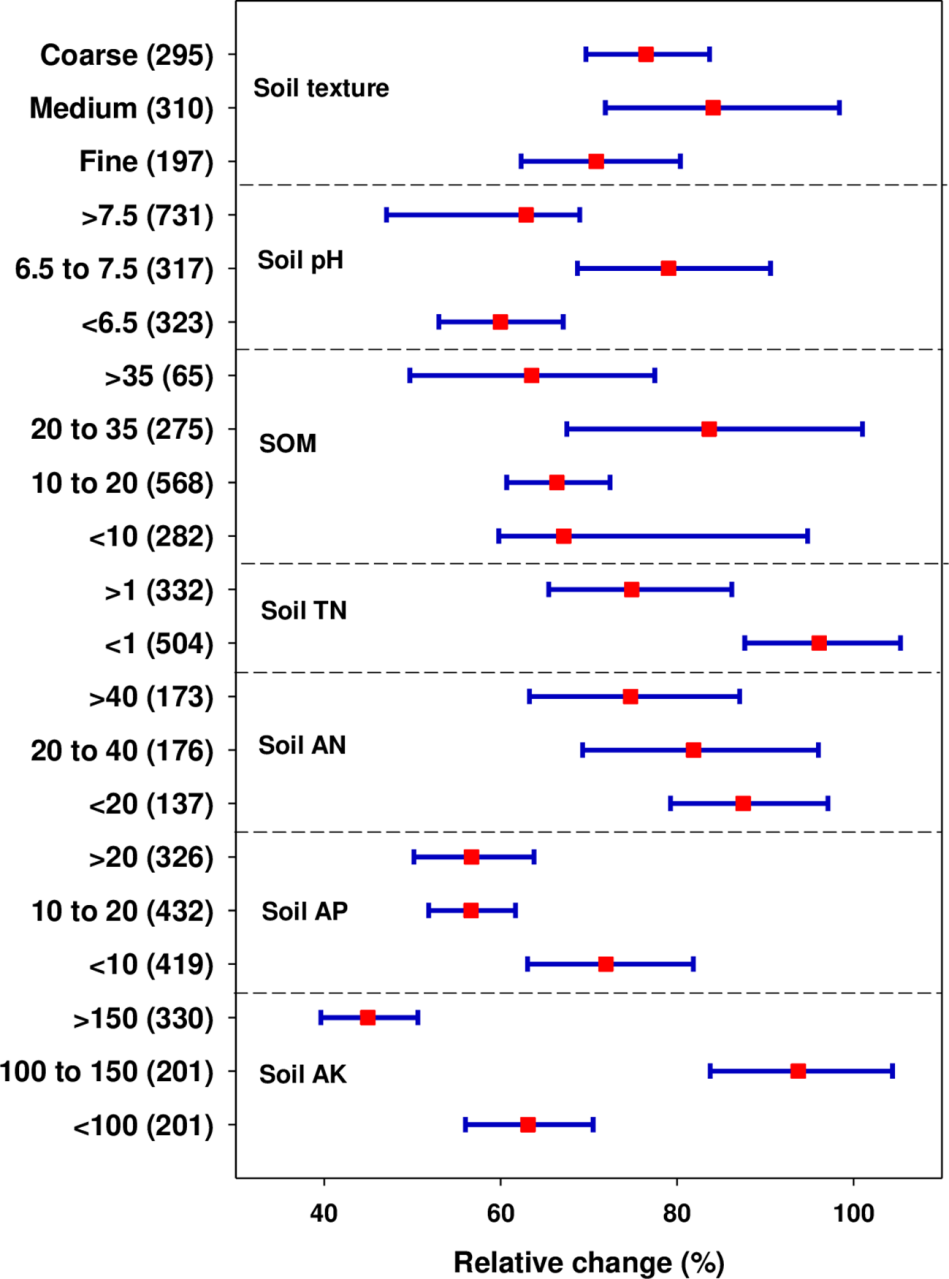
Effect of N fertilization on wheat yield as affected by initial soil properties (soil textural class, soil pH, initial available N, P and K (in mg kg^-1^), and initial soil organic matter (g kg^-1^). Error bars represent a mean value at 95% CIs. Numbers in the bracket represent observations.

Soil properties are one of the basic factors along with management and climate factors that determine the crop response to applied N fertilizer. The effects of initial soil properties are presented in **Figure 5**. The positive effect of N fertilization on yield was higher (79.04%) at pH 6.5-7.5 (neutral soil), whereas it remained at 59.96% and 62.9% at pH < 6.5 (acidic) and > 7.5 (alkaline) soils, respectively. A significantly positive yield response was obtained in all soil textural classes, but with varying magnitudes. The yield was significantly increased in medium-textured soils (84.08%) followed by coarse-textured soils (76.47%), however, the positive effect of N fertilization on wheat yield was reduced in fine-textured soils (70.83%) (**Figure 5**).

The positive effect of N fertilization on yield was not linear with SOM content (**Figure 5**). The response was greatest (83.64%) with SOM content between 20-35 g kg^-1^ but declined when SOM exceeded this range (63.52% with SOM > 35 g kg^-1^). Similarly, the positive effect of N fertilization on yield decreased with increased TN concentration. The yield increase with N application was significantly higher (96.1%) when TN was < 1 g kg^-1^ and lower (67.2%) with TN > 1 g kg^-1^. The positive effect of N fertilization on wheat yield showed a linear decline with the increase of initial AN concentration, being highest at AN< 20 mg kg^-1^ (87.49%) followed by AN between 20-40 mg kg^-1^ (81.88%) and low at AN > 40 mg kg^-1^ (74.73%). On the other hand, a more pronounced effect of N fertilization on yield was observed with AP < 10 mg kg^-1^ content (71.93%), but it reduced at AP contents between 10-20 and > 20 mg kg^-1^ (56.65 and 56.69%, respectively). Also, the positive response was greatest with AK contents between 100-150 mg kg^-1^ (93.74%), but it declined significantly with AK contents of < 100 mg kg^-1^ (63.1%) and > 150 mg kg^-1^ (44.94%) (**Figure 5**).

### 3.4 Wheat N recovery efficiency and affecting factors

**Figure 6:**
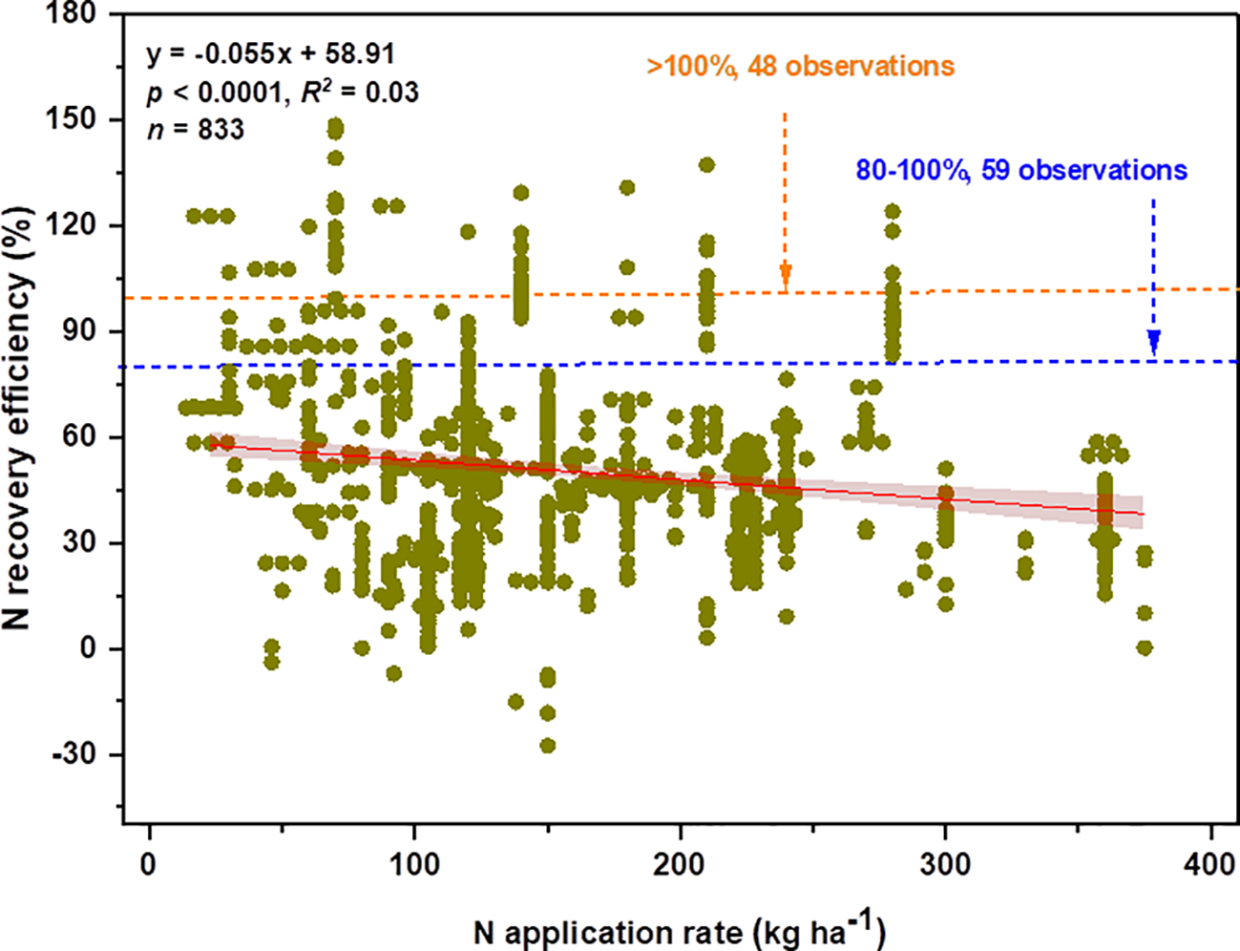
Nitrogen recovery efficiency (REN, in %) as a function of N application rate (kg ha^-1^). The blue and orange dashed lines represent the number of observations that had REN values between 80-100% and >100%, respectively.

**Figure 7:**
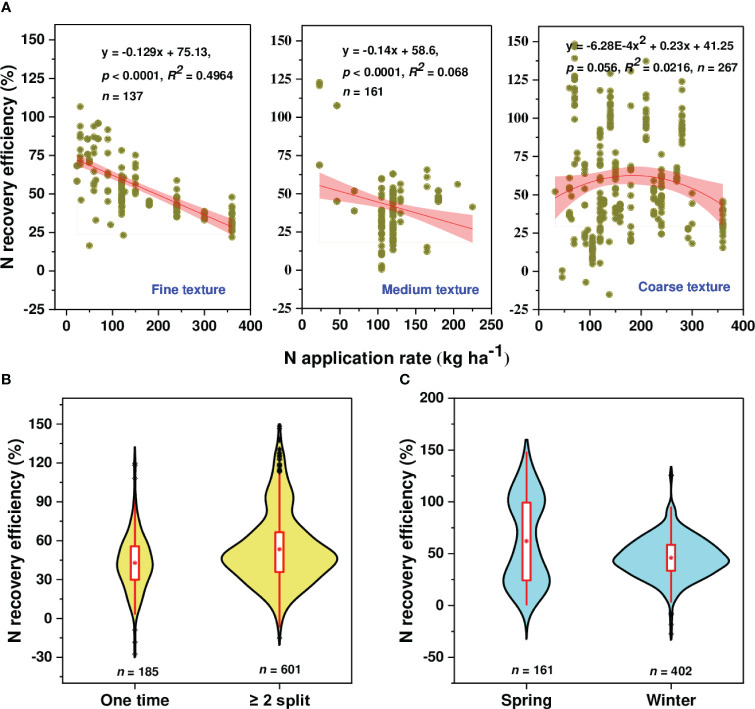
Relationships between N recovery efficiency and N application rate under different soil textural classes: **(A)** fine, medium and coarse-texture, **(B)** the violin plots of the effects of N fertilization on N recovery efficiency under different N timing (one-time and split) and **(C)** seasons (spring vs winter). A red dot in violin plot represents a mean value.

In this study, the recovery efficiency of N was defined as the difference between the amount of N uptake by plants grown with and without N fertilizer to fertilizer N inputs as indicated in equation (1). Across all observations (833 observations), the average REN was 49.78%, which showed a declining trend with an increasing N rate (**Figure 6**). The value of REN varied considerably among included observations ranging from -27.54 to 148.41%. Although most observations had a REN value below 80%, 59 and 48 observations had a REN value between 80-100% and >100%, respectively. We found a linear relationship between AE_N_ (kg kg^-1^) and PFP with REN (**Figure S5**). As PFP is described as the ratio of grain yield to applied fertilizer and agronomic efficiency as the ratio of yield advantage to N-application, thus a linear relationship observed with REN explained that an increase in REN was potentially driven by a yield increase. Moreover, the relationship between REN and N-application rate showed different trends depending on soil textural classes (**Figure 7A**). A linear decline of REN was observed with an N rate increase in medium and fine-textured soils and a quadratic decline in coarse-textured soils.

**Figure 8:**
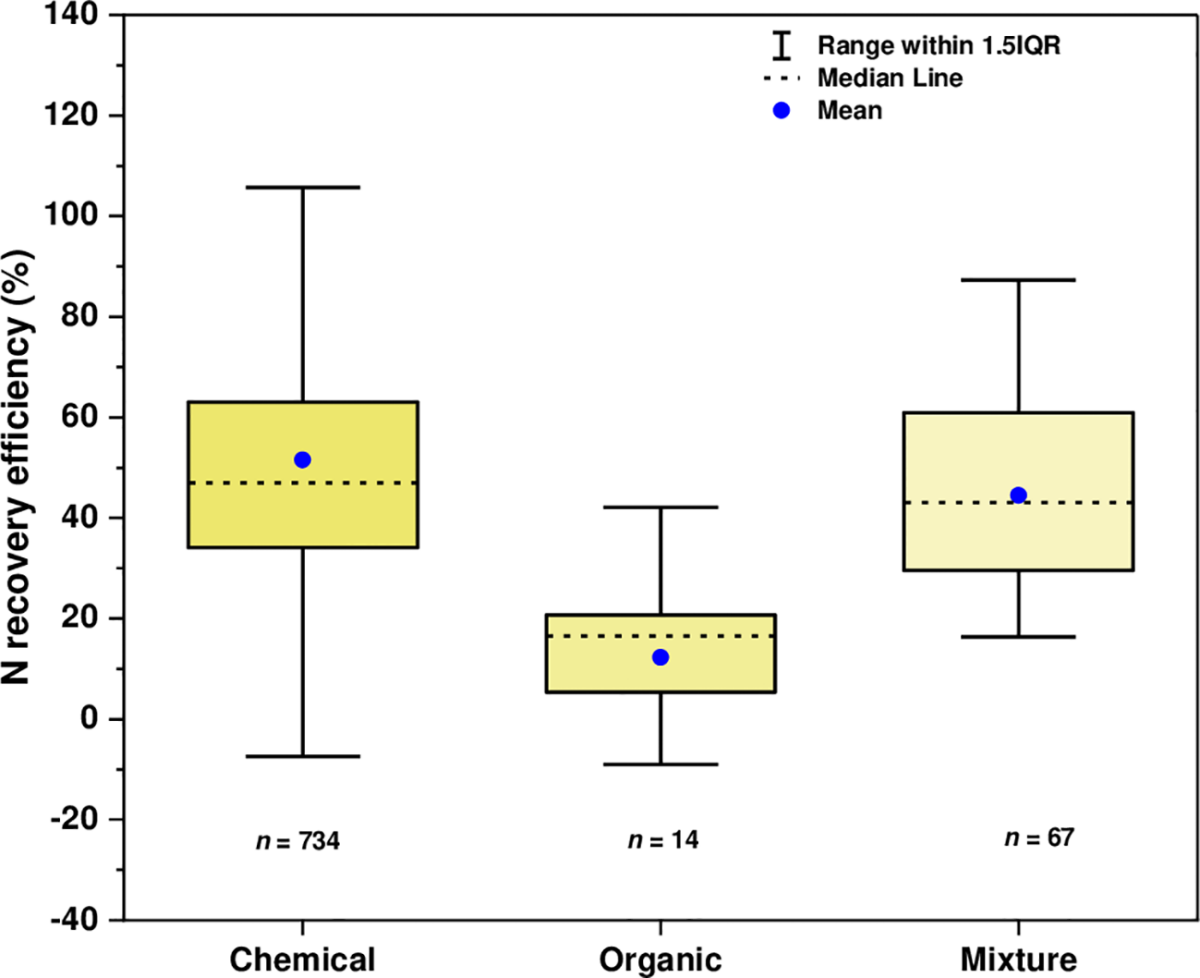
Nitrogen recovery efficiency in wheat crops as affected by N application sources (chemical, organic, and combined applications).

The value of REN was considerably varied depending on N timing/frequency and wheat type (**Figures 7B, C**), and N sources (**Figure 8**). The result revealed that when N was applied in split forms (≥ 2), REN was higher (53.39%) than when was applied once in a whole wheat growing season (42.85%) regardless of application rate. Moreover, the REN was more pronounced with spring wheat (62.165%) than with winter wheat (46.07%). Regarding N sources, we found higher REN with chemical sources (51.50%) followed by the combination of organic and chemical sources (44.46%), while it remained substantially lower with the sole application of organic sources (12.26%).

**Figure 9:**
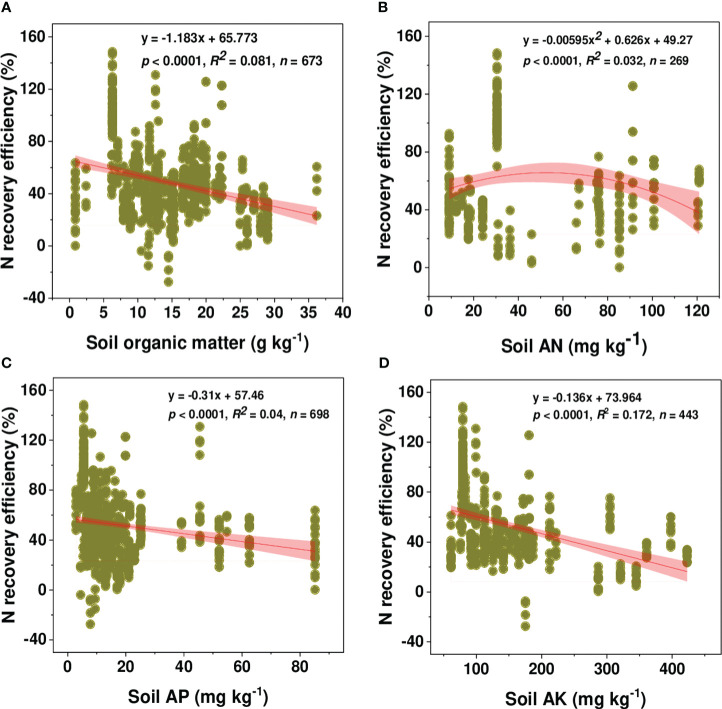
Relationship between wheat N recovery efficiency (%) and initial soil properties: **(A)** SOM, soil organic matter, **(B)** AN, available nitrogen, **(C)** AP, available phosphorous, and **(D)** AK, available potassium.

A regression analysis was conducted to confirm the possible relationship between REN and initial soil properties i.e., SOM, AN, AP, and AK (**Figure 9**). A linear regression model better explained the relationships between REN and initial soil properties of SOM, AP, and AK, but a quadratic model better fits REN and AN. SOM is one of the important factors for explaining crop productivity and thereby recovery efficiency. A regression model result showed that REN could significantly and linearly decrease with increasing SOM concentrations (y= -1.183x + 65.773, *p*< 0.0001, *n* = 673). Also, the relationship between REN and AP (y = -0.31x + 57.46, *p*< 0.0001, *n* = 698) and AK (y = -0.136x + 73.964, *p*< 0.0001, *n* = 443) showed a significant decline with the increase of concentration of these soil properties. On the other hand, a quadratic decline of REN was observed with the increase of initial AN concentration (y = -0.0059x^2 +^ 0.626x + 49.27, *p*< 0.0001, *n* = 269).

## 4 Discussion

### 4.1 Effect of management, climate and initial soil factors on N fertilization efficiency

Nitrogen is an important macro-nutrient for crop growth and development, while its deficiency in soil potentially affects crop productivity (Cheng et al., 2022a; Derebe et al., 2022). Being one of the major limiting nutrients, N is usually applied in agriculture to increase crop production, but excessive, inefficient, and imbalanced N application causes several environmental and economic impacts (Kubar et al., 2021; Song et al., 2022a). In this study, the highest wheat yield increase (81.68%) was observed at 150-300 kg ha^-1^ and a respective decline in yield (70.7%) with further addition of N fertilizer beyond this threshold amount as shown in (**Figures 2**, **3A**). Previous studies reported that with N levels up to 225 kg ha^-1^ a noticeable increase in wheat (Song et al., 2023) and maize (Jiang et al., 2019) productivity was observed regardless of N application methods; however, as N levels beyond this point, yields began to decline. The decrease in crop production with higher N fertilizer addition over the recommended amount may be attributed to exceed crop nutrient requirements, oversaturating plant nutrient absorption systems (Dobermann, 2005; Wang et al., 2011) and profoundly affecting the photosynthesis rate and grain filling potential in cereal crops thereby affecting grain yield. Also, excessive N supply may rapidly increase the biomass yield at the expense of grain yield, thus reducing grain yield. Moreover, in addition to having an impact on yield, it causes an excessive buildup of nitrate N (NO_3_^-^ N) in the soil, which causes large amounts of N loss to the environment from the soil-plant system (Chen et al., 2014; Cui et al., 2014), and resultantly affects crop productivity by reducing N availability in the soil. Rana et al. (2020) reported a linear relationship between N application rates and NO_3_^-^ leaching in the rice-wheat cropping system.

The fertilizer application method plays a significant role in overall nutrient efficiency, nutrient uptake, crop productivity, and gaseous emissions (Liu et al., 2015; Song et al., 2022a). In our meta-analysis, the effect of N fertilization on grain yield did not differ significantly between broadcasting and placement, but with incorporation (**Figure 2**). Banding of N under inadequate soil moisture may affect the nutrient movement in soil and N uptake potential, which ultimately contributes to lower grain yield. Also, surface broadcasting increases the risk of N loss through volatilization and runoff, and reduces available N for plant uptake (Song et al., 2023). However, the observed higher yield response with N incorporation is attributed to increased availability of N within the root zone and reduced leaching and run-offs, which thereby enhances N-uptake and grain yield. Guo et al. (2022) reported a higher yield response with fertilizer banding but showed non-significant differences with other N application modes.

Improper N application timing is an important yield-limiting factor of wheat crop production. Although the magnitude of yield response varied depending on split frequency (**Figure S3**), generally, split N application (*n* =1231) exhibited a substantially greater yield response than one-time application (*n* = 495) (**Figure 2**). The reason behind with higher yield response under split may be attributed to the better synchrony between the time of high need of plant N uptake and the availability of sufficient N in the soil at the specified growth stages (Haile et al., 2012). At the time of high need for N, the plant may have acquired most of the nitrate from the soil, leaving less of it available for leaching hence improving NUE. As the majority of a plant’s N uptake occurs at the later crop growth stage, so applying a large quantity of N at the early stage when the capacity of plant uptake is small can exacerbate N loss from the soil-plant system and contribute to low yield and recovery efficiency. Consistent with our findings, an earlier study reported a greater yield response when N was applied in three splits than one-time at tillering or even two times (Belete et al., 2018). Additionally, Derebe et al. (2022) reported that the yield of bread wheat increased as the number of N splitting increased from one to three. An increase in wheat yield (3.3%) was reported when N was applied in split than one-time, however, the magnitude of yield increase may be influenced by several explanatory factors such as N management practices, genotype, tillage practice, climatic and soil factors (Hu et al., 2021). Our result signifies that the combined application of chemical + organic N (**Figure 2**) as well as sole or combined application of release fertilizer with urea could profoundly influence grain yield (**Figure S4**). It is reported that the wheat crop requires a relatively large amount of N from the jointing to anthesis stages (Zheng et al., 2016). Thus, the low yield response with ammonium-based fertilizer application might be related to their tendency of rapid hydrolysis during the early growing stages when plants require less N and became insufficient when plants require high N at later growth stage. The continuous use of chemical fertilizer alone not only affects crop yield but also degrades SOM concentration and causes nutrient imbalance that leads to soil acidity (Bhatt et al., 2019). While organic inputs may not show a significant effect on crop yields, particularly within a short period of time (Wei et al., 2020; Zhang et al., 2020) due to nutrient immobilization and lower nutrient release patterns. The use of organic inputs alone may not be enough to maintain high crop yield due to its limited availability and relatively low nutrient content. Thus, the combined application of chemical and organic inputs has been suggested as a rational strategy not only to improve soil fertility and enhance crop production (Yang et al., 2015) but also in minimizing economic costs and reducing environmental problems. Regardless of the explanatory variables elucidated in this study, the substitution of synthetic N with manure enhanced crop yield while a full supply of manure reduced crop yield (Zhang et al., 2020).

Control/slow-release fertilizer synchronizes nutrient release and supply with crop nutrient uptake by slowing down the N release pattern and providing N to crops for a long period, thus reducing the risk of surplus N loss from the soil-plant system and enhancing crop yield and NUE (Yu et al., 2022b). It can minimize early season N availability when crop uptake is low, and increase N availability during the advanced stage when the N demand for crop is high. In line with our findings, an earlier study revealed that combined application of release fertilizer with conventional urea significantly enhances N availability for plant’s uptake, thereby increasing grain yield, and N recovery efficiency (33.7–56.4% for wheat crop) while reducing N leaching and saving labor and fertilizer costs (Zheng et al., 2016). Another study also confirmed that the integration of slow-release fertilizer and conventional urea enhances not only rice and wheat yields but also improves NUE in rice (27.4–96.5%) and wheat (22.8–57.1%) as compared to conventional urea alone (Yu et al., 2022b). However, the benefits and effectiveness of release fertilizers are affected by several factors such as soil moisture, temperature, coating materials, and mixing ratio, cropping patterns. Therefore, the potential of using release fertilizer in different areas must be assessed by considering the environmental conditions (Li et al., 2021).

In the present meta-analysis, spring wheat exhibited a significantly lower yield response (26.49%) than winter wheat (80.03%) (**Figure 4**). The lower yield response in spring wheat can be attributed to a combination of faster growth and development requiring higher N availability at early growth stages (Subedi et al., 2007). Likewise, the lower yield potential might be related to its shorter growth duration and high temperature during the growth stage (Wu et al., 2019). On the other hand, a higher grain yield of winter wheat was attributed to the production of a higher kernel number, kernel weight, and harvest index (Entz & Fowler, 1991). It can also be attributed to winter wheat having a deeper root system. According to a prior study (Thorup-Kristensen et al., 2009), the depth of winter wheat roots can be up to twice that of spring wheat roots. This increases the availability of water and reduces the possibility of N leaching. Our findings demonstrated that the response of wheat production to N treatment is considerably influenced by climatic factors. Temperature is an important factor influencing the rate of plant growth and productivity. Extreme temperatures (above or below the threshold value) at a critical plant growth stage can have a significant negative impact on productivity(Hatfield and Prueger, 2015). In this study, a significant yield response was 79.60% and 67.81% at MAT <15°C and >15°C, respectively (**Figure 4**). An earlier study revealed that an elevated temperature may increase nitrate concentration in the soil due to greater organic matter (OM) decomposition and gross N mineralization as a result of increased microbial metabolism and enzyme activity (Bai et al., 2013), thus reducing available soil N through high N loss as leaching and gas emission. According to Li et al. (2019), a significant decline in crop yields was revealed when MAT exceeds 15°C (i.e., 97.3 and 96.3% lower as compared with MAT 8–15 and <8°C, respectively). Likewise, an increase of a unit (1°C) warming resulted in an average decline of maize yield by 2.6%, which also varied with seasons (higher loss in summer maize than spring maize), indigenous soil properties (Deng et al., 2020), climatic factors and regional variations (Li et al., 2019; Deng et al., 2020), and N management practices and crop types (Zhou et al., 2021).

The distribution of rainfall across the seasons, both in terms of quantity and pattern, has a significant impact on crop response to N fertilization. Our analysis showed that regions receiving MAP >800 mm (88.99%) responded to N application more favorably than regions receiving MAP <800 mm (67.02%) (**Figure 4**). The increase of grain yield with MAP (> 800 mm) can be attributed to the importance of adequate precipitation during the wheat growing season which improves soil moisture contents and hence promotes mass flow and diffusion of soil N towards the wheat root system. However, caution must be applied because excessive precipitation may result in greater N loss primarily due to N leaching and runoff from agricultural fields. Recently, a greater yield response with high MAP under straw return conditions has been reported by (Islam et al., 2022). Despite they generate important information, the relation between yield response to N fertilization under different MAP and MAT ranges must be interpreted with caution. Due to insufficient reporting of mean growing season temperature and precipitation in our database, we used MAT and MAP instead. The reason is MAT and MAP may not be effective in revealing the real effect of climate conditions on crop yield and applied fertilizer at critical growth stages (Quan et al., 2021; Yokamo et al., 2022b).

The significance of soil to crop production and its quality is directly related to soil’s properties (Zhu et al., 2020). In this study, a wheat yield due to N fertilization is affected by soil basic properties. Soil pH is a key soil characteristic that plays a crucial role in the availability of soil nutrients, impacting plant nutrient uptake and use efficiency. It significantly affects the absorption and loss of administered N fertilizer by regulating soil microbial transformation. The higher yield response (79.04%) was observed in neutral soil (pH 6.5-7.5) (**Figure 5**). In neutral soils, most soil nutrients are optimally available for plants’ uptake. In low pH soils, the solubility of aluminum and manganese is high, which releases excess aluminum (Al^3+^) and manganese (Mn^2+^) ions in the soil which then affects plant N uptake and grain yield by influencing root growth and function. Al toxicity is the foremost factor in low-pH soils that retards crop production by impeding root growth and water and nutrient uptake (Szurman-Zubrzycka et al., 2021). This might be a reason for decreased yield response in acidic soils. The rise of pH (in the case of alkaline soil) not only exacerbates ammonia volatilization but also increases the nitrification-denitrification process (Quan et al., 2020), thus reducing soil N availability and resultantly crop yield. In concordant with our findings, greater crop yield response was reported in neutral soils (Guo et al., 2022).

A significantly positive yield response was obtained in all soil textural classes, though the magnitudes varied (i.e. high in medium-textured soils followed by coarse than fine-textured soils) (**Figure 5**). Fine-textured soils have maximum water-holding capacity, which alternatively produces anaerobic conditions in the soil, which exacerbates N loss from soil-plant systems. Also, it is vulnerable to surface compaction, especially during wet conditions, which confines soil infiltration and porosity. This further limits plant nutrient uptake and mobilization by impeding optimal root proliferation. This might be a reason for the relatively less yield response in fine-textured soils in this study. Shakoor et al. (2021) reported a significantly high and low grain yield in coarse and fine-textured soils, respectively.

Soil organic matter (SOM) is a key indicator of soil health and environmental quality owing to its important sink and the main nutrient source for the plant. Our meta-analysis revealed that the positive effect of N fertilization on yield was high with SOM content between 20-35 g kg^-1^, and declined when the SOM content exceeds this range as shown in **Figure 5**. A greater wheat and maize yield at SOC concentrations between 0.1-2.0% were reported by (Oldfield et al., 2019), but the yield increase remained consistent when the SOC contents exceeded 2.0%. Another study reported a decline in yield with split N application at higher SOM and nitrate-N contents (Hu et al., 2021). Similarly, the positive effect of N fertilization on yield decreased with increased TN, AN, AP, and AK concentrations in soil. Overall, our findings revealed a high yield response in medium-textured and neutral soils (pH 6.5-7.5), and soils having low-medium fertility status. Similar results have been reported by earlier studies (Wang et al., 2020; Yokamo et al., 2022b).

### 4.2 Key factors affecting N recovery efficiency in wheat crop

An overarching goal of agronomic research has been to understand how to regulate N inputs and pinpoint the procedures that maximize N recovery. In both large-scale and small-scale systems across the world, achieving the synchrony between N supply and plant N requirements is essential for maximizing a trade-off between agronomic, economic, and environmental quality. Thus, for sustainable food production and environmental quality, a quantitative understanding of the fates of fertilizer N (present levels of N-use efficiency and potential loss) as well as its control mechanisms is essential (Cassman et al., 2002). Our present study revealed that the average global wheat REN was 49.78%, with an increasing N rate showing a declining trend (**Figure 6**), demonstrating the enormous potential for REN improvement (Quan et al., 2021). In the rice-wheat cropping system, Rana et al. (2020) observed an increase in NUE up to a certain level with an increasing N rate, which revealed a negative association with a continuous supply of N. Previously, the global NUE (calculated in N-difference approach) in eight cereal crops was documented at 35% (Omara et al., 2019) and 39% for three major crops of wheat, rice, and maize (Yu et al., 2022a). Only 47% of the reactive N added globally onto cropland been recovered in the past three decades, which is considerably low as compared with NUE in the early 1960s (68%) even though the usage of chemical N expanded by a factor of nine during this time (Lassaletta et al., 2014).

Proper N application timing and rates are critical for meeting crop’s N requirement (Haile et al., 2012). In the current study, split N application resulted in a higher REN than a single application during a whole wheat growing season (**Figure 7B**), revealing the potential of split N application in REN improvement for wheat crops. The reason might be a pre-plant N may be subject to leaching and prone to denitrification or immobilization before plants’ active uptake stage, thus affecting NUE, while the split application may enhance the synchrony of N supply with wheat N demands, which thereby enhances grain yield and NUE (Subedi et al., 2007). Moreover, yield improvement coupled with reduction of unproductive N loss with split N application enhanced REN. The majority of reactive nitrogen losses through emission, leaching, and runoff are known to occur during the early stages of crop growth, when root N uptake is least efficient. Moreover, the REN was more pronounced with spring wheat (62.165%) than with winter wheat (46.07%) as shown in **Figure 7C**. This is mainly attributed to a higher N application rate in winter wheat than spring wheat as REN is the function of grain yield and N-rate (on average, 169.95 and 119.25 kg N ha^-1^ were applied for winter and spring wheat, respectively).

However, we obtained a reduced REN with sole organic N supply as opposed to chemical or combined application (**Figure 8**) because the use of a carbon substrate in conjunction with organic fertilizers may encourage denitrification, leading to increased nitrogen loss and decreased recovery efficiency. In contrast with the result we obtained in **Figure 2** for productivity, the highest REN was revealed with synthetic N supply followed by their combination with organic N inputs. The efficacy of REN has not been sustained with further improvement in soil fertility. As shown in **Figure 9**, a linear regression analysis (R^2^ between 0.032-0.172) showed a declining trend with a further increase in initial soil properties, showing that the addition of N to already fertile soils may affect plant uptake and recovery efficiency. Most of the observations in our study came from short-term studies, thus, we have not taken into account the legacy effect of long-term N application. For instance, long-term application of synthetic or organic inputs may increase soil nutrient pools, which further affects plants’ response to the applied fertilizer. It is believed that soil N availability may increase with experimental duration and continuous N supply; under such circumstances, plants rely less on the applied N during the crop growing season which might reduce REN (Yu et al., 2022a).

## 5 Summary and future perspectives

The findings of the present meta-analysis showed an overall increase in wheat yield (62.81%) with N application. In addition, the global average REN for the wheat crop remained at 49.78%. The variation in yield response depends greatly on N management practices, climate factors, and initial soil factors. More importantly, the wheat yield and recovery efficiency remained higher with N split application than applying one time during a growing season regardless of application methods and frequency. However, a growing literature reveals the impossibility of mediating the challenges that the world is facing, such as unprecedented growth of food demand and environmental pollution with only split application. For instance, the split N application involves additional farm operations that increase labor requirements, fuel consumption, and CO_2_ emissions (Hu et al., 2021). Although wheat yield increased with the number of N-split applications, grain protein has declined at various sites (Derebe et al., 2022). While the potential of plant N-uptake is also dependent on moisture levels, a lack of precipitation can seriously impair the movement of N in the root zone and its subsequent uptake by plants through inhibited root growth. Therefore, it is not a convenient method for reducing potential N loss and environmental pollution while simultaneously increasing yield and NUE. Hence, more decisive N management methods that optimally match N supply and crop N demand, improve crop productivity and NUE, while reducing environmental pollution, and have economic benefits are needed urgently.

**Figure 10:**
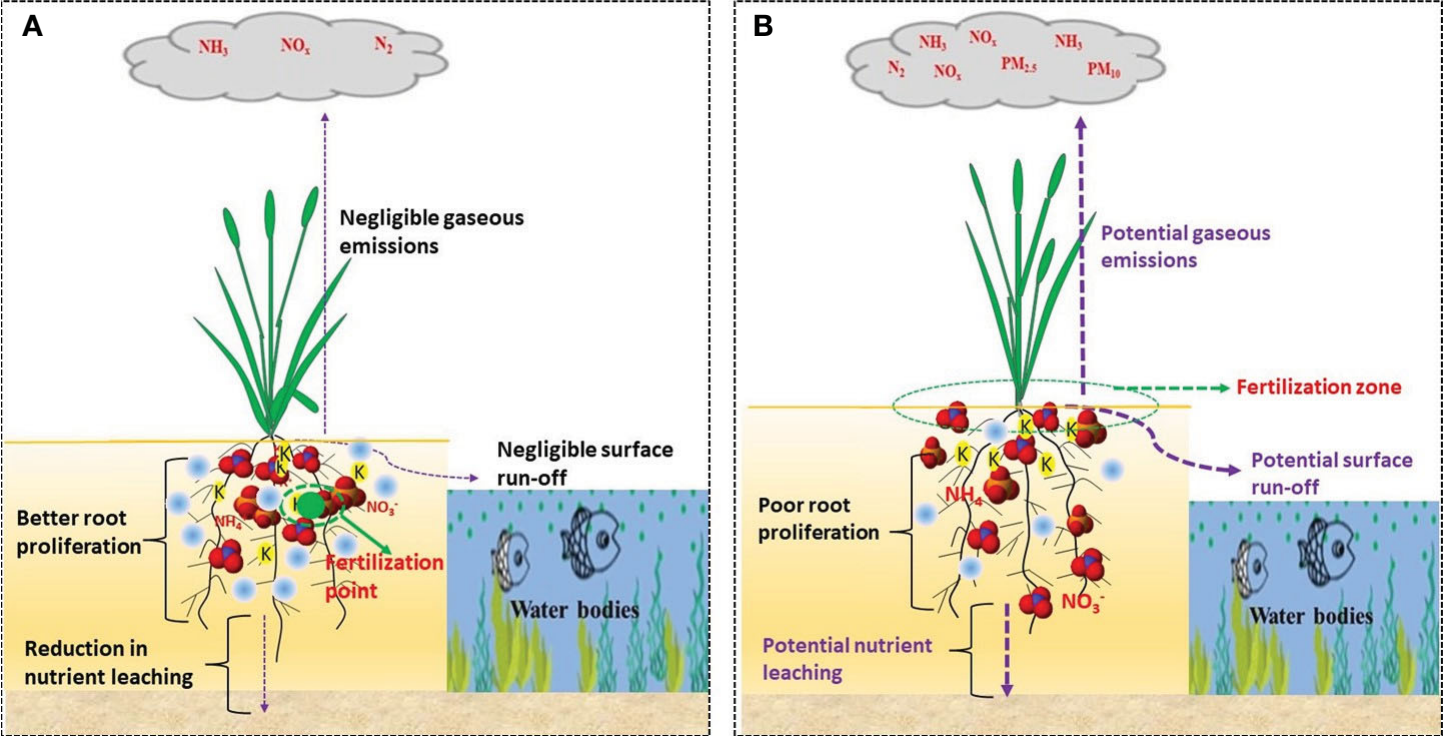
Schematic representations of **(A)** one-time root-zone targeted fertilization and **(B)** conventional surface broadcasting nutrient management practices. The size of the purple arrow represents the magnitude of nutrient losses through gaseous emission, leaching and run-off.

Adopting a precise fertilizer placement method in the root zone, where crops still have a substantial amount of moisture is getting more credit than split surface application (**Figure 10**). The point, root zone targeted fertilization (P-RZTF) has been proposed by Wang and Zhou (2013). Supplying all the fertilizer at once during the whole plant growth period is a promising method that can replace traditional fertilizer (particularly N) application methods (Jiang et al., 2019). The RZTF refers to an exact deep, and point application of all the fertilizers just once in the root-zone during the whole plant growing season. It is capable of reducing the N application rate without penalizing grain yield due to its potential to supply an adequate amount of nutrients for plant uptake. Moreover, it allows the applied N fertilizer to be distributed in the active root zone, which then enhances root proliferation and promotes N uptake by the root system (Jiang et al., 2019). Application of N in this method can prolong N retention and reduce potential loss by influencing ammonia-oxidizing archaea and bacteria (Cheng et al., 2023). Studies conducted at field levels through this technique revealed promising results on yield, NUE, or gaseous emissions such as maize (Jiang et al., 2018, 2019; 2022), rice (Lu et al., 2019; Song et al., 2022a), wheat (Chen et al., 2016; Song et al., 2022b), and oilseed rape (*Brassica Napus* L.) (Cheng et al., 2022b), against a conventional split and broadcasting methods. More importantly, the negligible ammonia volatilization (which is nearly close to the control plot) was reported in a transplanted rice field with RZTF technique (Song et al., 2022a). Overall, RZTF has the potential to offset the conundrum that comes from split and broadcasting, and other conventional N fertilization techniques while improving yield and NUE, minimizing reactive N loss, and increasing economic gains by saving not only N application rate but also intensive labor force. As RZTF has a broad application prospect, it should be further optimized and by combining not only with a suitable placement machinery but also with other innovative strategies such as integrating novel fertilizer products and soil conditioners and/or developing big-particle fertilizers that suits for ease of application. In summary, our result revealed a positive yield response to N fertilization, but several affecting factors including N management strategies (optimal combinations of fertilizer rate, time, sources, and placement locations) should be adequately planned to ensure green and sustainable production in the future.

## Data availability statement

The datasets presented in this study can be found in online repositories. The names of the repository/repositories and accession number(s) can be found in the article/**Supplementary Material**.

## Author contributions

SY: Conceptualization, Data curation, Formal Analysis, Methodology, Visualization, Writing – original draft. MIr: Data curation, Methodology, Software, Writing – review & editing. WH: Investigation, Software, Data curation, Writing – review & editing. BW: Investigation, Software, Writing – review & editing. YW: Investigation, Software, Writing – review & editing. MIs: Data curation, Methodology, Software, Writing – review & editing. DL: Investigation, Writing – review & editing. XC: Investigation, Writing – review & editing. QC: Funding acquisition, Supervision, Writing – review & editing. HW: Funding acquisition, Supervision, Validation, Writing – review & editing.
