# Supplementary material for: Global evaluation of key factors influencing nitrogen fertilization efficiency in wheat: a recent meta-analysis (2000-2022)
Source: Front Plant Sci. 2023 Oct 30;14:1272098. doi: 10.3389/fpls.2023.1272098 (PMC10642427; doi:10.3389/fpls.2023.1272098)
Supplement: Supplementary file 1 [file DataSheet_1.docx]

Supplementary Material

**This file includes:**

Figures S1, S2, S3, S4 and S5;

Table S1


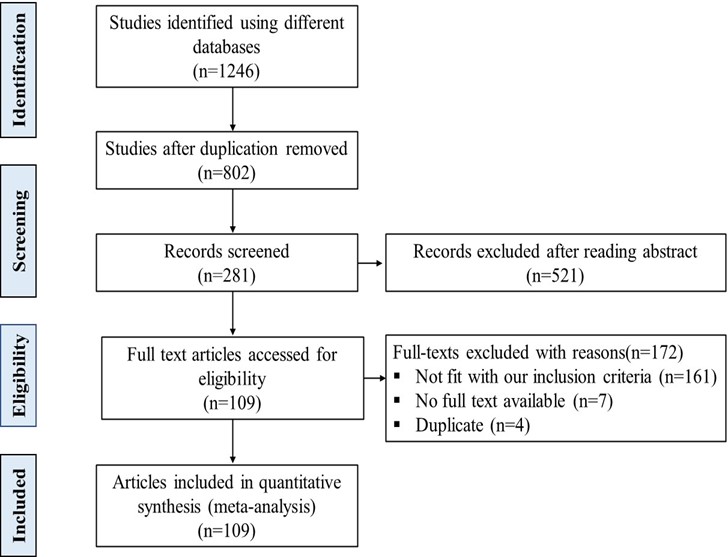


**Figure S1.** Flowchart diagram of the process applied for the meta-analysis

**
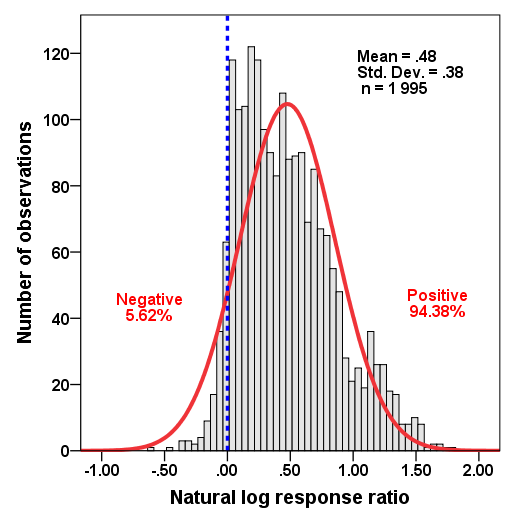
**

**Figure S2.** Histogram of natural log response ratio (ln*RR*) of wheat yield response to N fertilization at the global level (*n* = 1995). The blue dotted line represents a line of zero effect.

**
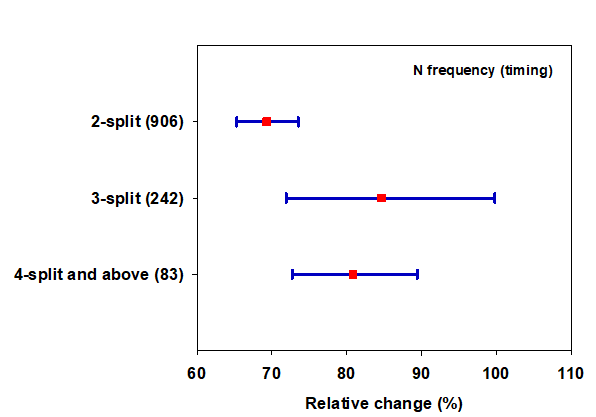
**

**Figure S3.** Effect of N fertilization on wheat yield as affected by frequency of N split. Error bars represent a mean value at 95% CIs. Numbers in parentheses indicate the number of observations.

**
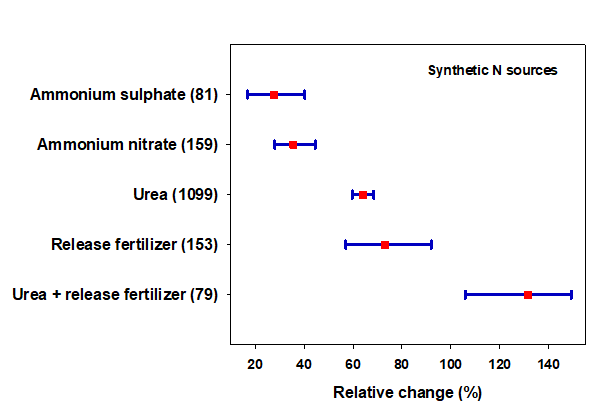
**

**Figure S4.** Effect of N fertilization on wheat yield as affected by different chemical N sources. Error bars represent a mean value at 95% CIs. Numbers in parentheses indicate the number of observations.


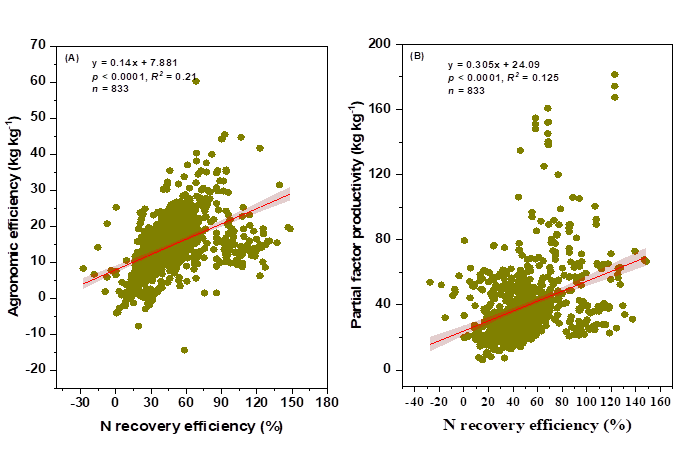


**Figure S5.** Relationship between agronomic N use efficiency (kg kg^-1^) **(A)** and partial factor productivity (kg kg^-1^) **(B)** with N recovery efficiency (%) in wheat crop

**Table S1.** Bibliography for studies included in the meta-analysis.

| Author-year | Title of the journal | Country |
| --- | --- | --- |
| Abril et al. 2007 | Effect of wheat crop fertilization on nitrogen dynamics and balance in the Humid Pampas, Argentina | Argentina |
| Wallace et al. 2018 | Fertiliser timing and use of inhibitors to reduce N_2_O emissions of rainfed wheat in a semi-arid environment | Australia |
| Haque et al. 2017 | Response of yield, nitrogen use efficiency, and grain protein content of wheat (*Triticum aestivum l.*) Varieties to different nitrogen levels | Bangladesh |
| Timsina et al. 2001 | Cultivar, nitrogen, and water effects on productivity and nitrogen-use efficiency and balance for rice-wheat sequences of Bangladesh | Bangladesh |
| Benin et al. 2012 | Agronomic performance of wheat cultivars in response to nitrogen fertilization levels | Brazil |
| Asgedom et al. 2016 | Nitrous oxide emissions from a clay soil receiving granular urea formulations and dairy manure | Canada |
| Grant, Moulin and Tremblay 2016 | Nitrogen management effects on spring wheat yield and protein concentration vary with seeding date and slope position | Canada |
| Duan J et al. 2019 | Optimizing nitrogen management to achieve high yield, high nitrogen efficiency and low nitrogen emission in winter wheat | China |
| Guo et al. 2019 | Root and nitrate-N distribution and optimization of n input in winter wheat | China |
| Ma et al. 2019 | Determining the optimal N input to improve grain yield and quality in winter wheat with reduced apparent N loss in the North China Plain | China |
| Wang et al. 2022 | Effects of nitrogen application rate under straw incorporation on photosynthesis, productivity and nitrogen use efﬁciency in winter wheat | China |
| He et al. 2013 | Effect of nitrogen management on productivity, nitrogen use efficiency and nitrogen balance for a wheat-maize system | China |
| Khan et al. 2020 | Soil water consumption, water use efficiency and winter wheat production in response to nitrogen fertilizer and tillage | China |
| Zhang et al. 2017 | Effect of irrigation and nitrogen application on grain amino acid composition and protein quality in winter wheat | China |
| Qiao et al. 2022 | Effect of continuous N fertilizer reduction on N losses and wheat yield in the Taihu Lake region, China | China |
| Zhu et al. 2011 | Enhancing nitrogen use efficiency by combinations of nitrogen application amount and time in wheat | China |
| Xu et al. 2020 | Exploring optimal irrigation and nitrogen fertilization in a winter wheat-summer maize rotation system for improving crop yield and reducing water and nitrogen leaching | China |
| Yi et al. 2018 | Is there a nitrogen fertilizer threshold emitting less N_2_O with the prerequisite of high wheat production? | China |
| Zheng et al. 2016 | Combining controlled-release urea and normal urea to improve the nitrogen use efﬁciency and yield under wheat-maize double cropping system | China |
| Xu et al. 2021 | Use of controlled-release urea to improve yield, nitrogen utilization, and economic return and reduce nitrogen loss in wheat-maize crop rotations | China |
| Jing-xiu et al. 2021 | Yield performance and optimal nitrogen and phosphorus application rates in wheat and Faba bean intercropping | China |
| Li et al. 2021 | Split application of a mixture of controlled-release and common urea for improving quality and agronomic and economic performance in wheat production | China |
| Li R et al. 2021 | Blended controlled-release nitrogen fertilizer with straw returning improved morphology of wheat soil nitrogen availability, soil microbial community, and root morphology of wheat |  |
| Geng et al. 2016 | Controlled release urea improved nitrogen use efficiency and yield of wheat and corn | China |
| Tao et al. 2021 | A 2-year study of the impact of reduced nitrogen application combined with double inhibitors on soil nitrogen transformation and wheat productivity under drip irrigation | China |
| Shi et al. 2021 | Assessing the impacts of biochar-blended urea on nitrogen use efficiency and soil retention in wheat production | China |
| Wang et al. 2016 | Suppression of ammonia volatilization from rice-wheat rotation fields amended with controlled release urea and urea | China |
| Liu et al. 2021 | Impacts of nitrogen practices on yield, grain quality, and nitrogen-use efﬁciency of crops and soil fertility in three paddy-upland cropping systems | China |
| Liu and Huang 2016 | The effect of Nitrogen Rates on yields and Nitrogen use efficiency during Four Years of Wheat-maize Rotations Cropping Seasons | China |
| Qiang et al. 2020 | Nitrogen application affects grain yield by altering the soil moisture and nitrate-N of maize/wheat cropping system in dryland areas of northwest China | China |
| Chen et al. 2022 | Different organic material amendments effects soil nitrogen utilization and crop yield in the North China Plain | China |
| Yang et al. 2011 | Controlled release urea improved nitrogen use efﬁciency, yield, and quality of wheat | China |
| Wang et al. 2012 | Nitrogen fertilization effect on soil water and wheat yield in the Chinese loess plateau | China |
| Zheng et al. 2017 | Improving crop yields, nitrogen use efﬁciencies, and proﬁts by using mixtures of coated controlled-released and uncoated urea in a wheat-maize system | China |
| Yu et al. 2022 | Effect of combined application of slow-release and conventional urea on yield and nitrogen use efﬁciency of rice and wheat under full straw return | China |
| Shakoor et al. 2017 | Effects of fertilizer application schemes and soil environmental factors on nitrous oxide emission fluxes in a rice-wheat cropping system, East China. | China |
| Zhao and & Si 2015 | Effects of topdressing with nitrogen fertilizer on wheat yield, and nitrogen uptake and utilization efficiency on the Loess Plateau | China |
| Peng et al. 2017 | Responses of nitrogen utilization and apparent nitrogen loss to different control measures in the wheat and maize rotation system | China |
| Shi et al. 2012 | Effects of nitrogen applications on soil nitrogen balance and nitrogen utilization of winter wheat in a rice-wheat rotation | China |
| Liu et al. 2003 | Nitrogen dynamics and budgets in a winter wheat–maize cropping system in the North China Plain | China |
| Xue, Yu and Yang 2014 | Maintaining yields and reducing nitrogen loss in rice–wheat rotation system in Taihu Lake region with proper fertilizer management | China |
| Min-hua, Yuan-nong and Yuan-bo 2017 | Comparative effects of nitrogen application on growth and nitrogen use in a winter wheat/summer maize rotation system | China |
| Liu et al. 2018 | Root growth, water and nitrogen use efﬁciencies in winter wheat under different irrigation and nitrogen regimes in North China Plain | China |
| Yi et al. 2015 | Optimizing fertilizer nitrogen for winter wheat production in Yangtze River region in China | China |
| Liang et al. 2008 | The ecologically optimum application of nitrogen in wheat season of rice–wheat cropping system | China |
| Li et al. 2021 | Long-term effects of straw mulching coupled with N application on soil organic carbon sequestration and soil aggregation in a winter wheat monoculture system | China |
| Yao et al. 2013 | Greenhouse gas ﬂuxes and NO release from a Chinese subtropical rice-winter wheat rotation system under nitrogen fertilizer management | China |
| Xue-yun, Ben-hua and Shu-lan 2014 | Trends of yield and soil fertility in a long-term wheat-maize system | China |
| Cui et al. 2022 | One-Time nitrogen fertilizer application using controlled-release urea ensured the yield, nitrogen use efﬁciencies, and proﬁts of winter wheat | China |
| Mansour et al. 2017 | Nitrogen use efficiency in spring wheat: genotypic variation and grain yield response under sandy soil conditions | Egypt |
| Belete et al. 2018 | Effect of split application of different N rates on productivity and nitrogen use efficiency of bread wheat (Triticum aestivum L.) | Ethiopia |
| Nano 2017 | Influence of nitrogen fertilizer application on grain yield, nitrogen uptake efficiency, and nitrogen use efficiency of bread wheat (triticum aestivum l.) Cultivars in Eastern Ethiopia | Ethiopia |
| Amante et al. 2014 | Optimum NP fertilizers rate for wheat production on alfisols of Arjo and Shambu Highlands, Western Ethiopia | Ethiopia |
| Fresew et al. 2018 | Effect of nitrogen fertilizer rates on grain yield and nitrogen uptake and use efficiency of bread wheat (Triticum aestivum L.) varieties on the vertisols of central highlands of Ethiopia | Ethiopia |
| Melesse 2015 | Response of bread wheat (Triticum aestivum L.) varieties to N and P fertilizer rates in Ofla district, Southern Tigray, Ethiopia | Ethiopia |
| Bekalu and Mamo 2016 | Effect of the rate of n-fertilizer application on growth and yield of wheat (Triticum aestivum l.) at Chencha, southern Ethiopia | Ethiopia |
| Bekalu and Arega 2016 | Effect of the time and rate of N-fertilizer application on growth and yield of wheat (triticum aestivum l.) At Gamo-gofa Zone, Southern Ethiopia | Ethiopia |
| Alemu, Sharma and Firdissa 2016 | Effect of weed management practices and nitrogen levels on weeds, yield components and yield of bread wheat (triticum aestivum L.) at Kulumsa, Southeastern Ethiopia | Ethiopia |
| Bizuwork and Yibekal 2020 | Optimizing blended (NPSB) and N fertilizer rates for the productivity of Durum wheat (Triticum turgidum L.var. durum) in Central Highlands of Ethiopia | Ethiopia |
| Alemu, Ketema and Tesfaye 2019 | Response of Wheat (Triticum aestivumL.) to different rates of nitrogen and phosphorus at Fiche-Salale, Highlands of Ethiopia | Ethiopia |
| Hassen et al 2022 | Effects of irrigation levels and nitrogen fertilizer rate on grain yield of Wheat (Triticum aestivum) at Amibara, Middle Awash, Ethiopia | Ethiopia |
| Sofonyas, Lemma and Selamyihun 2020 | Nitrogen use efficiency, yield and yield traits of wheat response to slow-releasing N fertilizer under balanced fertilization in Vertisols and Cambisols of Tigray, Ethiopia | Ethiopia |
| Wogene and Agena 2017 | Response of bread wheat varieties to different levels of nitrogen at Doyogena, Southern Ethiopia | Ethiopia |
| Woyema, Bultosa and Taa 2012 | Effect of different nitrogen fertilizer rates on yield and yield related traits for seven Durum wheat (Triticum Turgidum L. Var Durum) cultivars grown at Sinana, South Eastern Ethiopia | Ethiopia |
| Beyenesh and Nigussie 2017 | Performance of bread wheat (Triticum aestivum L.) in response to supplemental irrigation and rate of nitrogen application in Enderta, Tigray, Northern Ethiopia | Ethiopia |
| Bereket et al. 2014 | Effects of mineral nitrogen and phosphorus fertilizers on yield and nutrient utilization of bread wheat (Triticum aestivum) on the sandy soils of Hawzen District, Northern Ethiopia | Ethiopia |
| Koutroubas et al. 2016 | Effect of organic manure on wheat grain yield, nutrient accumulation and translocation | Greece |
| Sandhu et al. 2019 | Drip irrigation and nitrogen management for improving crop yields, nitrogen use eﬃciency and water productivity of maize-wheat system on permanent beds in north-west India | India |
| Singh and Dwivedi 2006 | Yield and nitrogen use efficiency in wheat, and soil fertility status as influenced by substitution of rice with pigeon pea in a rice–wheat cropping system | India |
| Upadhayay and Kaushal 2021 | Effect of organic and inorganic source of nitrogen on yield of wheat (Triticum aestivum L.) | India |
| Shivay et al. 2017 | Response of spring wheat to boron-coated urea and its effect on nitrogen use efficiency | India |
| THIND et al. 2010 | Managing neem (Azadirachta indica)-coated urea and ordinary urea in wheat (Triticum aestivum) for improving nitrogen-use efficiency and high yields | India |
| Yadvinder-Singh et al. 2009 | Nitrogen and residue management effects on agronomic productivity and nitrogen use efﬁciency in rice–wheat system in Indian Punjab | India |
| Sandhu et al. 2020 | Evaluation of N fertilization management strategies for increasing crop yields and nitrogen use eﬃciency in furrow-irrigated maize–wheat system under permanent raised bed planting | India |
| Behera, Pradhang and Sharma 2007 | Effect of integrated nutrient management practices on productivity of durum wheat (Triticum durum) in the Vertisols of central India | India |
| Balamurugan, Lakshmanan and Ramasamy 2021 | Wheat growth, yield, and yield contributing attributes as a function of nitrogen Levels | India |
| Abedi et al. 2011 | Wheat yield and grain protein response to nitrogen amount and timing | Iran |
| Efretuei et al. 2016 | Effect of nitrogen fertilizer application timing on nitrogen use efficiency and grain yield of winter wheat in Ireland | Ireland |
| Guarda, Padovan and Delogu 2004 | Grain yield, nitrogen-use efﬁciency and baking quality of old and modern Italian bread-wheat cultivars grown at different nitrogen levels | Italy |
| Giambalvo et al. 2010 | Nitrogen use efﬁciency and nitrogen fertilizer recovery of durum wheat genotypes as affected by interspeciﬁc competition | Italy |
| Nakano, Morita and Kusuda 2008 | Effect of nitrogen application rate and timing on grain yield and protein content of the bread wheat cultivar ‘Minaminokaori’ in Southwestern Japan | Japan |
| Shahzad and Akmal 2016 | Yield performance of wheat under split n application rates and timing | Pakistan |
| Abbasi and Tahir 2012 | Economizing nitrogen fertilizer in wheat through combinations with organic manures in Kashmir, Pakistan | Pakistan |
| Shah et al. 2010 | Effect of integrated use of organic and inorganic nitrogen sources on wheat yield | Pakistan |
| Shah et al. et al 2009 | N uptake and yield of wheat as influenced by integrated use of organic and mineral nitrogen | Pakistan |
| Ali et al. 2011 | Effects of nitrogen on growth and yield components of wheat | Pakistan |
| Khan et al. 2022 | Eﬀect of diﬀerent nitrogen rates and split applications on growth and productivity of wheat cultivars | Pakistan |
| Rehim et al. 2020 | Integrated use of farm manure and synthetic nitrogen fertilizer improves nitrogen use efficiency, yield and grain quality in wheat | Pakistan |
| Ullah et al. 2018 | Effect of different nitrogen levels on growth, yield and yield contributing attributes of wheat | Pakistan |
| Hameed et al. 2003 | Effects of different planting dates, seed rate and nitrogen application level on wheat | Pakistan |
| Iqibal, Hayat and Hussain 2012 | Effects of nitrogen and plant density on maize (zea mays l.) Phenology and grain yield | Pakistan |
| Gorjanović et al. 2009 | Effect of nitrogen rate on grain yield of bread wheat genotypes | Serbia |
| Kostic´ et al. 2021 | The Effect of N fertilizer application timing on wheat yield on chernozem soil | Serbia |
| López-Bellido and López-Bellido 2001 | Efficiency of nitrogen in wheat under Mediterranean conditions: effect of tillage, crop rotation and N fertilization | Spain |
| Lo´pez-Bellido, Castllo and Lo´pez-Bellido 2008 | Comparative response of bread and durum wheat cultivars to nitrogen fertilizer in a rainfed Mediterranean environment: soil nitrate and N uptake and efﬁciency | Spain |
| Lo´pez-Bellido et al. 2000 | Effects of tillage, crop rotation, and nitrogen fertilization on wheat under rainfed Mediterranean conditions | Spain |
| Garrido-Lestache, Lo´pez-Bellido and Luis Lo´pez-Bellido 2004 | Effect of N rate, timing and splitting and N type on bread-making quality in hard red spring wheat under rainfed Mediterranean conditions | Spain |
| Lo´pez-Bellido, J. Lo´pez-Bellido, Redondo 2005 | Nitrogen efﬁciency in wheat under rainfed Mediterranean conditions as affected by split nitrogen application | Spain |
| Ali Tahir et al. 2020 | Genetic gain in wheat grain yield and nitrogen use efficiency at different nitrogen levels in an irrigated hot environment | Sudan |
| Adams et al. 2018 | Agronomic and economic effects of two enhanced-efficiency urea fertilizer technologies on Southern Great Plains winter wheat | USA |
| Walsh et al. 2022 | Spring wheat yield and grain quality response to nitrogen rate | USA |
| Walsh et al. 2020 | Grain yield, quality, and spectral characteristics of wheat grown under varied nitrogen and irrigation | USA |
| Mohammed et al. 2013 | Nitrogen fertilizer management for improved grain quality and yield in winter wheat in Oklahoma | USA |
| Walsh and Christiaens 2016 | Relative efficacy of liquid nitrogen fertilizers in dryland spring wheat | USA |
| Savala, Crozier and Smyth 2016 | Poultry manure nitrogen availability influences winter wheat yield and yield components | USA |
| Ghimire et al. 2021 | Effects of cultivars and nitrogen management on wheat grain yield and protein | USA |
| Lin et al. 2019 | Double-Crop wheat and soybean yield response to poultry litter application | USA |
| Dhillon et al. 2020 | Nitrogen management impact on winter wheat grain yield and estimated plant nitrogen loss | USA |
| Gaudin et al. 2015 | Wheat improves nitrogen use efﬁciency of maize and soybean-based cropping systems | USA |
